# Glutamate dehydrogenase hyperinsulinism: mechanisms, diagnosis, and treatment

**DOI:** 10.1186/s13023-023-02624-6

**Published:** 2023-01-31

**Authors:** Qiao Zeng, Yan-Mei Sang

**Affiliations:** 1 Department of Anesthesiology, The Children’s Hospital, Zhejiang University School of Medicine, Hangzhou, 310052 China; 2 Department of Endocrinology, Genetics and Metabolism Centre, Beijing Children’s Hospital, Capital Medical University, National Centre for Children’s Health, Beijing, 100045 China

**Keywords:** Congenital hyperinsulinism (CHI), Glutamate dehydrogenase hyperinsulinism (GDH-HI), Glutamate dehydrogenase 1 (*GLUD1*) gene

## Abstract

Congenital hyperinsulinism (CHI) is a genetically heterogeneous disease, in which intractable, persistent hypoglycemia is induced by excessive insulin secretion and increased serum insulin concentration. To date,15 genes have been found to be associated with the pathogenesis of CHI. Glutamate dehydrogenase hyperinsulinism (GDH-HI) is the second most common type of CHI and is caused by mutations in the glutamate dehydrogenase 1 gene. The objective of this review is to summarize the genetic mechanisms, diagnosis and treatment progress of GDH-HI. Early diagnosis and treatment are extremely important to prevent long-term neurological complications in children with GDH-HI.

## Introduction

**Fig. 1:**
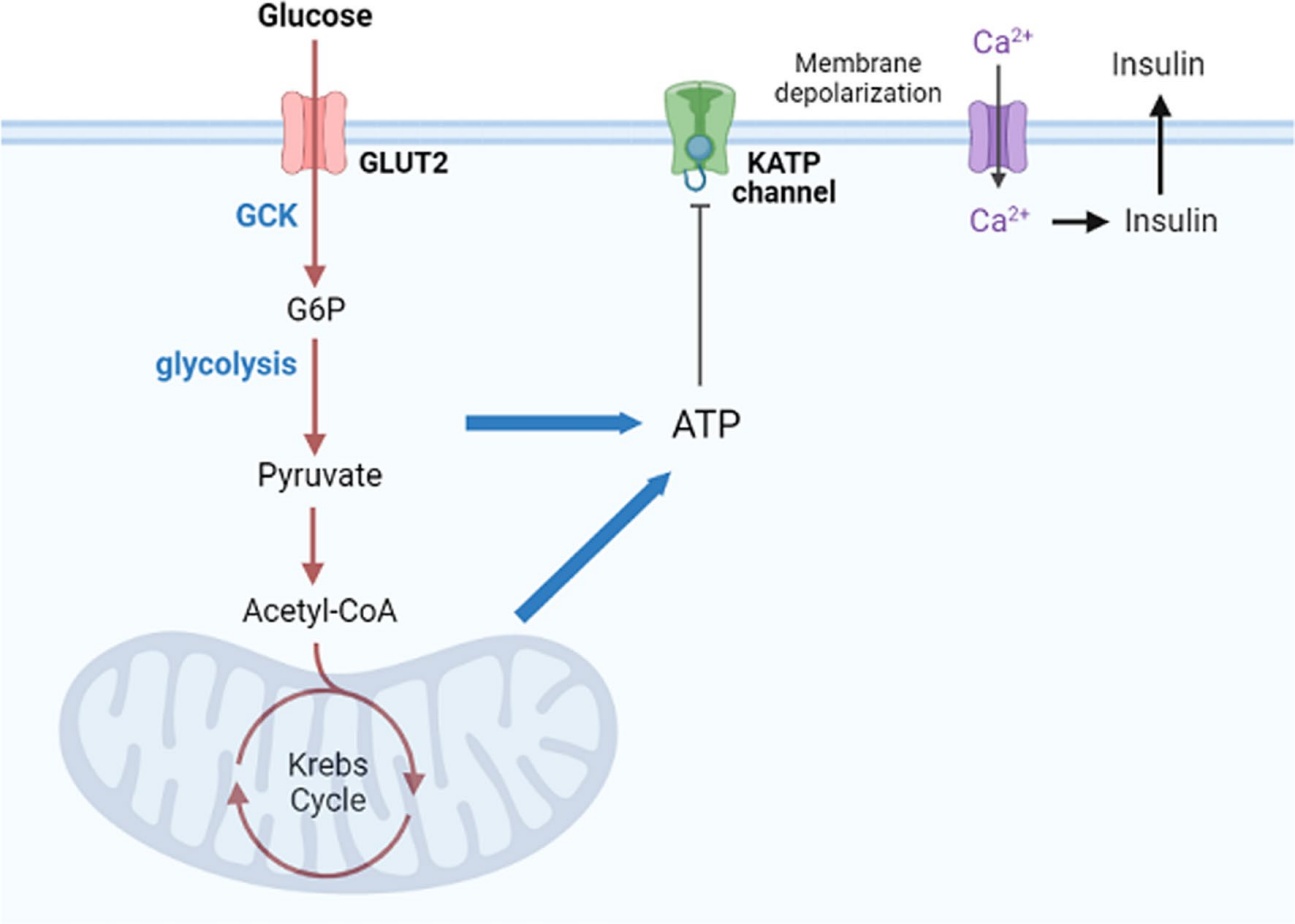
Extracellular glucose and insulin maintain the glucose homeostasis pathway. GLUT2, glucose transporter 2; GCK, glucokinase; G6P, generating glucose-6-phosphate

In the pancreatic beta cell, extracellular glucose concentration is transduced to ATP/ADP ratio, and it is ultimately the ATP/ADP ratio that determines insulin release. In islet β cells, extracellular glucose is transported into the cytoplasm by glucose transporter 2 (GLUT2), where glucose is subsequently phosphorylated by glucokinase (GCK), generating glucose-6-phosphate (G6P). G6P then generates ATP through glycolysis, tricarboxylic acid (TCA) cycle, and oxidative phosphorylation. Intracellular ATP binds to the Kir6.2 subunits of the ATP-sensitive potassium (K_ATP_) channel and inhibit its activity, causing the closure of K_ATP_ channel, further leading to β-cells membrane depolarization and voltage-gated calcium channel opening, resulting in calcium ion influx. The influx of calcium ions promotes the fusion of insulin-secreting vesicles with the cell membrane, leading to enhanced insulin secretion, which maintains glucose homeostasis [1] (Fig. 1). Glutamate dehydrogenase hyperinsulinism (GDH-HI) is induced by mutations in the glutamate dehydrogenase 1 (GLUD1) gene, which through several pathways lead to augmentation of insulin release that is independent of extracellular glucose concentration.

Congenital hyperinsulinism (CHI) is the leading cause of persistent, recurrent hypoglycemia in newborns and infants. CHI is a genetically and histologically heterogeneous disease marked by an inappropriately elevated level of insulin in the setting of underlying hypoglycemia. To date, 15 pathogenic genes associated with CHI have been identified, representing 14 genetic types.

Glutamate dehydrogenase hyperinsulinism (GDH-HI) is the second most common type of CHI and also known as hyperinsulinism/hyperammonemia (HI/HA) syndrome and familial hyperinsulinemic hypoglycemia-6 (HHF6) (OMIM # 606,762), accounting for about 5–13% of all CHI cases [2]. To date, more than 100 GDH-HI cases have been reported worldwide. This article reviews the genetic mechanism and progress in the diagnosis and treatment of GDH-HI, with a view to improving clinicians' understanding of GDH-HI.

## An introduction glutamate dehydrogenase physiology

In humans, glutamate dehydrogenase (GDH) is found in the mitochondrial matrix and is mainly expressed in the pancreas, liver, kidney, and brain. The functional GDH is a hexamer structure composed of two sets of trimers, with each trimer containing ~ 500 amino acids and each monomer possessing a guanosine triphosphate (GTP) binding site and a nicotinamide adenine diphosphate hydride (NADH)/adenosine diphosphate (ADP) binding site, enabling binding of 6 GTP molecules, 6 ADP molecules and 6 NADH molecules with GDH hexamer at its respective allosteric binding sites. The two trimers of GDH are intertwined by a substrate, and the subunits bind each other via a protruding antennae-like domain [3].

In animals, GDH is allosterically regulated by a variety of small molecules. The main allosteric inhibitors are GTP and ATP. GTP bind to the allosteric domain located above the catalytic domain at the bottom of the antenna of GDH, disabling GDH catalytic activity and preventing the release of oxidative products of glutamate, which is a rate-limiting step. NADH can bind to the NADH/ADP binding site and enhance the inhibitory effect of GTP on GDH activity by slowing down the release of NAD(P)H, an oxidative product of glutamate. The NADH/ADP binding site is located behind the catalytic cleft below the GDH pivot helix [3]. In addition, palmitoyl-CoA, steroid hormones and diethylstilbestrol (DES) are also inhibitors of mammalian GDH, but their binding sites are unknown. Epigallocatechin gallate (EGCG), a polyphenol in green tea, is also an allosteric inhibitor that competes with ADP [4].

The primary allosteric activators of GDH are ADP and leucine. ADP acts in the opposite way of GTP. ADP activates GDH by binding to the NADH/ADP binding site, and thus abrogating GTP binding. Leucine is a catalytic substrate for GDH and an allosteric activator of GDH as well. Its activation mechanism is similar to ADP, but has a different binding site [4].

### GDH function in organs

Under normal conditions, GDH is a key regulator for amino acid and ammonia metabolism in the human pancreas, liver, and brain. GDH also plays an important regulatory role in the stimulation of insulin secretion by amino acids (especially leucine). GDH uses NAD(P)^+^ as a coenzyme to catalyze the oxidative deamination of glutamate to produce alpha-ketoglutaric acid (α-KG) and ammonia. This reversible reaction is as follows: Glutamate + NAD(P)^+^  ↔ α-KG + NH_3_ + NAD(P)H. α-KG is used in the tricarboxylic acid (TCA) cycle to produce adenosine triphosphate (ATP). In islet β, brain, and renal tubular cells, the reaction is mainly directed towards oxidative deamination of glutamate due to high glutamate and low α-KG/NH_3_ levels [5].

In islet β cells, increased intracellular ATP/ADP ratio leads to closure of KATP channels and an influx of calcium ions, resulting in insulin secretion. The silent information regulator 4 (Sirtuin 4, SIRT4) can inhibit the activity of GDH in β-cells, thereby inhibiting insulin secretion [5]. SIRT4 is an ADP-ribose transferase located in mitochondria and is mainly expressed in the pancreas, brain, liver, and heart. In mitochondria, SIRT4 regulates energy metabolism, such as regulating the TCA cycle by downregulating the activity of GDH and malonyl coenzyme A decarboxylase (MCD). The activity of GDH is inhibited by ADP-ribosylation of the C172 residue at the active site of GDH. SIRT4 catalyzes ADP-ribosylation of GDH using NAD^+^ as a substrate, thereby reducing the activity of GDH. However, the exact reaction mechanism is unclear. Studies have shown [6] that SIRT4 can inhibit fatty acid oxidation in hepatocytes and muscle cells, therefore, GDH may be involved in lipid metabolism through SIRT4.

**Fig. 2:**
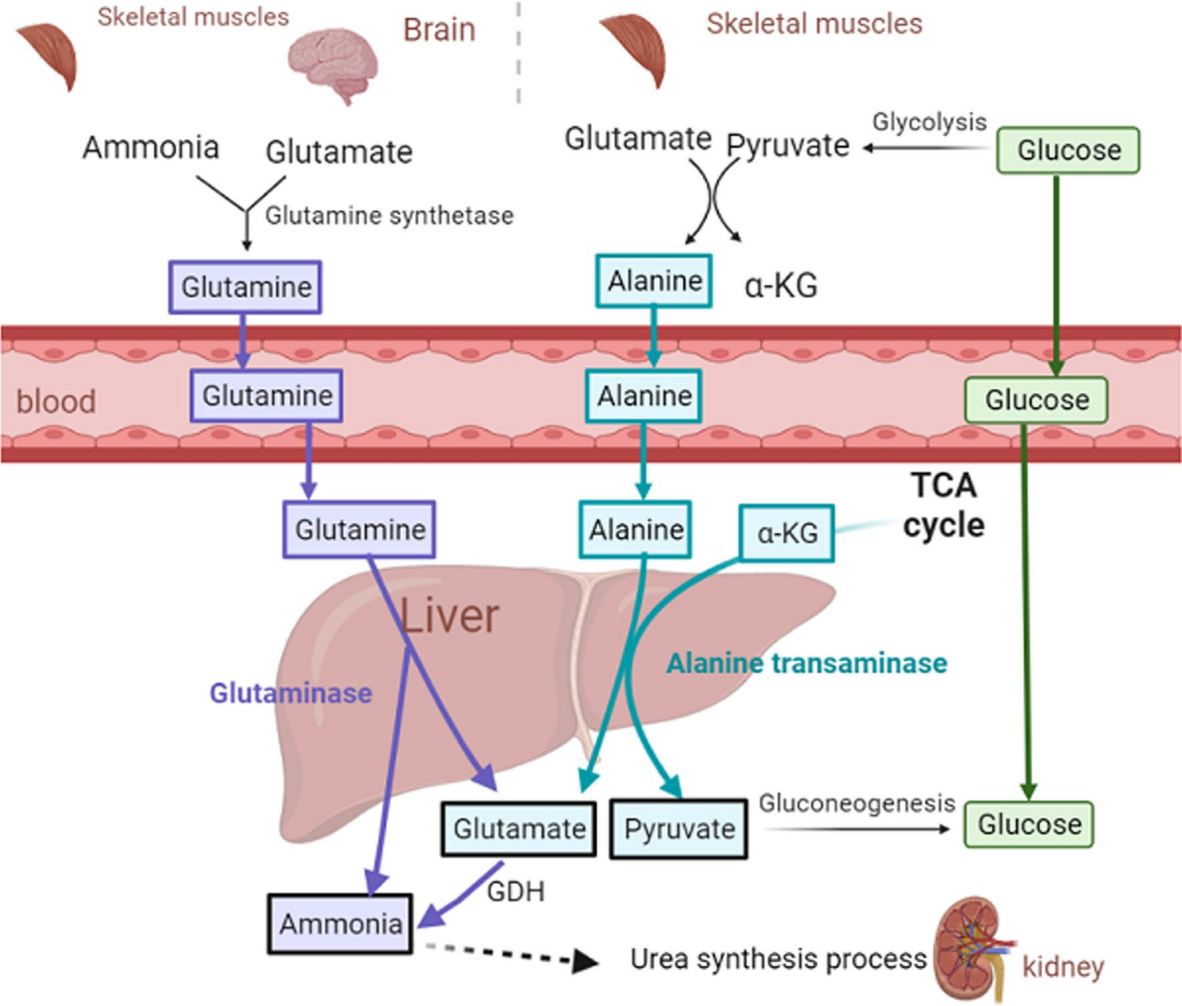
Ammonia produced by amino acid decomposition is transported in two ways: 1) in the form of glutamine. Under the catalyzation of glutamine synthetase (GS), the reaction of ammonia and glutamate produces glutamine, which is transported to the liver through blood, and decomposed to glutamate and ammonia via catalyzation by glutaminase. The ammonia then enters the urea synthesis process; 2) in the form of alanine. Pyruvic acid produced by glycolysis in muscles reacts with ammonia via the catalyzation of transaminases, producing alanine. Alanine is transported to the liver through blood and reacts with α-KG to produce pyruvic acid and glutamate via catalyzation by alanine transaminase. Glutamate produced in both ways is deaminated and the generated ammonia enters the urea synthesis process and is further removed from the body. α-KG, alpha-ketoglutaric acid; TCA cycle, tricarboxylic acid cycle; GDH, glutamate dehydrogenase

In hepatocytes, GDH constitutes ~ 1% of total proteins. The enzyme activity of GDH in hepatocytes is 4.5 times that of other tissues. Glutamate in the hepatocytes is involved in the metabolism of most amino acids through transamination. Therefore, the hepatocytes is the center of nitrogen metabolism in the human body. Figure 2 demonstrates the transport and metabolism of ammonia in the form of glutamine and alanine respectively. Renal ammonia genesis is primarily via glutamine metabolism, in which GDH plays an important role [7].

## Introduction to GLUD1, the gene that encodes GDH

The human GDH cDNAs was first cloned and published by Nakatani et al. [8]. In 1998, Stanley CA et al. had presented the first clear genotype–phenotype association between mutations of mitochondrial glutamate dehydrogenase, encoded by GLUD1 gene, and HI/HA syndrome [9]. *GLUD1*(NM_005271.4) is located on chromosome 10q23.3 with a length of 45 kb containing 13 exons, and 12 introns and encodes 505 amino acids [10]. To date, at least 39 mutations were identified in *GLUD1-*associated HI. The inheritance of GDH-HI is mainly autosomal dominant or secondary to de novo mutations, of which autosomal dominant inheritance accounts for ~ 20% while de novo mutations account for ~ 80% of all cases [11]. Studies on the molecular structure of GDH show that these mutations are closely related to GTP binding regulatory sites (Table 1).

**Table 1:** Reported mutated types of GLUD1 gene in GDH-HI patients

Exons	Gene mutation	Protein variation	References
6	c.809C > G	p.S270C	Trana et al. [32]
6	c.820C > T	p.R274C(p.R221C)	Roy et al. [38]
6	c.822C > G	p.S217C	Macmullen et al. [11]
7	c.943C > T	p.H315Y	Faletra et al. [39]
7	c.955 T > C	p.Y319H	Faletra et al. [39]
7	c.965C > T	p.R322H(p.R269H)	Xu et al. [20]
7	c.966G > A	p.R265K(p.R318K)	Kapoor et al. [21]
7	c.966G > C	p.R265T	Macmullen et al. [11]
7	c.969A > G	p.Y266C	Macmullen et al. [11]
7	c.977C > T	p.R269C	Macmullen et al. [11]
7	c.1046A > C	p.E349A(p.E296A)	ClinVar
7	c.1058G > C	p.E296Q	Bahi-Buisson et al. [18]
10	c.1387A > T	p.N463Y	Faletra et al. [39]
10	c.1388A > T	p.N463I	Sang et al. [31]
10	c.1388A > G	p.N463S	Sang et al. [31]
10	C20.1400A > G	p.N410D	Kapoor et al. [21]
10	c.1401A > C	p.N410T	Kapoor et al. [21]
10	c.1401A > T	p.N410I	Fang et al. [13]
10	c.1396 T > G	p.L413V	David et al. [19]
11	c.1479C > T	p.P436L	Kapoor et al. [21]
11	c.1486A > T	p.R443W	Ninković et al. [2]
11	c.1492C > A	p.F440L	Stanley et al. [40]
11	c.1494A > G	p.Q441R	Stanley et al. [40]
11	c. 1493C > T	p.S498L (p.S445L)	Barrosse-Antle et al. [41]
12	c.1493G > C	p.G446A	Stanley et al. [40]
12	c.1495C > A	p.G499C (p.G446C)	Sang et al. [31]
12	c.1496G > T	p.G499V	Luczkowska et al. [42]
12	c.1498G > A	p.A500T	Faletra et al. [39]
12	c.1508G > C	p.G446R	Stanley et al. [40]
12	c.1508G > A	p.G446S (p.G499S)	Stanley et al. [40]
12	c.1509G > A	p.G446D(p.G499D)	David et al. [19]
12	c.1509G > T	p.G446V	Stanley et al. [40]
12	c.1511G > A	p.A447T	Stanley et al. [40]
12	c.1514 T > C	p.S448P(p.S501P)	David et al. [19]
12	c.1516G > A	p.V453M	David et al. [19]
12	c.1519G > A	p.H507Y(p.H454Y)	Sang et al. [31]
12	c.1520A > G	p.K503E (p.K450E)	David et al. [19]
12	c.1524A > T	p.D451V	Kapoor et al. [21]
12	c.1526G > C	p.G509A	Trana et al. [32]

## Genetic mechanisms of GDH-HI

Mutations in *GLUD1* were first discovered in exons 11 and 12. Exons 11 and 12 encode the antenna-like domain, which is located at the pivot helix of the GDH structure and adjacent to the GTP allosteric binding domain encoded by exons 6 and 7. Mutations in these regions alter the GDH antenna-like domain and weaken the binding capacity of GTP binding domains with GTP, thereby compromising the inhibitory effect of GTP on GDH activity. Stronger GDH activity results in a higher ATP/ADP ratio and increased insulin secretion, leading to the onset of GDH-HI [2].

As more studies on GDH-HI were carried out, more mutations were identified in exons 6, 7, and 10. Exons 6 and 7 encode the GTP allosteric binding domain in GDH, which is the main binding site for GTP. Mutations in these regions can disturb GTP binding and lead to decreased sensitivity of GDH to allosteric regulation by GTP, resulting in weakened inhibitory effect and enhanced GDH activity [12].

Exon 10 encodes the α-helix of GDH, which locates on the other side of the hinge region. Studies showed that p.N410I, p.L413V, and p.N410T, three gene mutations in exon 10, can increase the activity of GDH (about twice the normal value) [13, 14]. Specific mechanisms regarding how these mutations regulate the activity of GDH are unclear.

### Mechanism of hypoglycemia development in GDH-HI patients

**Fig. 3:**
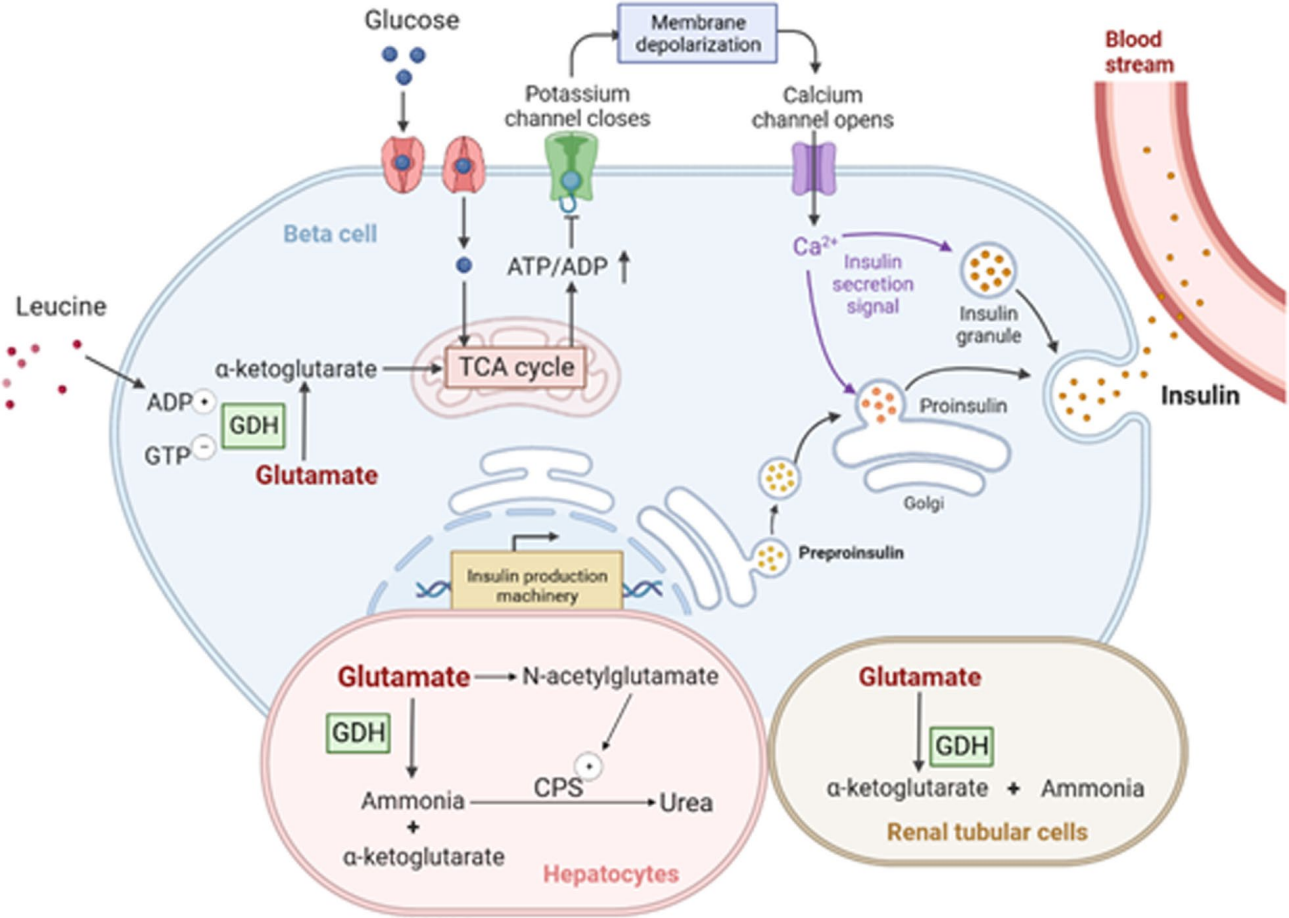
Mechanism of GDH mediated hypoglycemia/hyperammonemia in GDH-HI. In the beta cells, oxidation of glucose and activation of GDH by leucine increase the ratio of ATP/ADP, which leads to the closure of ATP-dependent potassium channels, the depolarization of cell membrane, the influx of calcium ions, and insulin secretion. In the hepatocytes, glutamate can produce ammonia catalyzed by GDH. Glutamate also generates N-acetylglutamate, an allosteric activator of CPS, to regulate ammonia detoxification into urea. In the renal tubular cells, GDH catalyzes glutamate to produce α-ketoglutarate and ammonia. GDH, glutamate dehydrogenase; TCA cycle, tricarboxylic acid cycle; CPS, carbamoyl phosphate synthetase

GDH catalyzes the oxidative deamination of glutamate to produce α-KG and ammonia. Overactive GDH can lead to decreased sensitivity to GTP, leading to increased α-KG levels due to glutamate dehydrogenation. More α-KGs enter the TCA cycle and more ATPs are generated, resulting in increased intracellular ATP/ADP ratio, followed by K_ATP_ channel closure, membrane depolarization, calcium ion influx, and excessive insulin secretion (Fig. 3). Leucine can allosterically activate GDH. Thus, a protein diet can increase the oxidation of glutamate and the ratio of ATP/ADP, causing increased insulin secretion. Therefore, due to the loss of inhibitory control of GDH by mutations in *GLUD1*, insulin was secreted excessively under basal conditions as well as after protein meals, triggering hyperinsulinemia and hypoglycemia [15].

Studies showed that the insulin-independent mechanism is another reason for fasting hypoglycemia in GDH-HI. During the hypoglycemic period after a meal in GDH-HI patients, the plasma insulin concentrations may be reduced, indicating that high insulin levels are not the only cause of hypoglycemia in GDH-HI patients. In 2014, Richard G. Kibbey et al. conducted a study on humanized mice carrying pathogenic p.H454Y mutations. Their results showed that mitochondria guanosine triphosphate (mtGTP) in islet β cells was insensitive, inhibiting the secretion of glucagon by α cells and leading to glucagon deficiency. This study showed for the first time that GDH-induced hypoglycemia was associated with impaired function of α cells [12]. Impaired glucagon secretion is a contributing mechanism.

Except for Leucine-mediated ATP production, other important mechanisms lead to the accumulation of citrate, leading to SCHAD-augmented insulin release [16]. α-KG produced by GDH increases the production of pyruvate, oxaloacetate and acetyl-CoA in mitochondria. Oxaloacetate and acetyl-CoA are used to produce citrate by the action of citrate synthase. Pyruvate supplies oxaloacetate to citrate synthase via pyruvate carboxylase reaction. In addition, theα-KG can be converted to succinyl-CoA, which is also an activator of GDH [16].

### Mechanism of hyperammonemia in patients with GDH-HI

In hepatocytes, elevated GDH activity leads to an increased rate of glutamate decomposition to ammonia. Excessive oxidation of GDH leads to a decrease of glutamate in hepatocytes, which in turn reduces the production of N-acetylglutamate (NAG), an allosteric activator of carbamoyl phosphate synthetase (CPS). CPS, on the other hand, is an essential allosteric activator for CPS-I in the urea synthesis process and also regulates the urea synthesis rate (Fig. 3). Therefore, urea synthesis is greatly reduced, increasing the free ammonia [2].

In 2010, Treberg et al. [7] demonstrated that increased renal ammoniagenesis may be the primary cause of hyperammonemia in GDH-HI patients. This study found that in animals treated with 2-Aminobicyclo [2, 2, 1] heptane-2-carboxylic acid (BCH), a nonmetabolic analogue of leucine, the renal arteriovenous ammonia level change was greater than in the liver, indicating that hyperammonemia in GDH-HI patients is mainly induced due to increased renal ammonia production, instead of abnormalities in urea synthesis in the liver. This could also explain why α-KG levels increase in the urine of patients, which is also a diagnostic marker for HI [7].

In the liver, kidney, and brain, in addition to catalyzing glutamate deamination to produces α-KG and ammonia, GDH can also catalyze deamination in other amino acids, such as L-leucine, L-2-aminobutyric acid, and L-valine.

### The pathogenesis of neurological abnormalities in GDH-HI patients

In the brain, glutamate is one of the most important excitatory neurotransmitters in the central nervous system (CNS). Glutamate neurotransmission has important effects on synaptic activity associated with normal nervous system development, learning, and memory formation. In glutaminergic neurons, glutamine is catalyzed by glutaminase (GLS) to synthesize glutamate, which binds to certain receptors to form excitatory potential. Intersynaptic glutamate uptake by astrocytes is the most important way to maintain the stability of extracellular glutamate concentration. Glutamate uptake by astrocytes is converted to glutamine by glutamine synthetase (GS). Subsequently, glutamine is released and ingested by glutamatergic neurons or γ-aminobutyric acid (GABA) neurons. In GABA neurons, glutamic acid decarboxylase (GAD) catalyzes glutamate to produce GABA, which binds to GABA receptors and generates inhibitory potentials [17]. Glutamate/GABA imbalance can cause nerve excitotoxicity, leading to nerve cell damage and neurological dysfunction.

In addition, glutamate can be oxidized through conversion to α-KG, a process catalyzed by GDH or transaminase. GDH catalyzed oxidative deamination is the main process for glutamate to enter the TCA cycle in the form of α-KG [6].

The main neurological abnormalities in GDH-HI patients are epilepsy, others include learning disability, cognitive impairment, behavioural problems, and delayed developmental milestones. The exact pathophysiological mechanism is unclear. Most patients did not display abnormalities during neuroimaging [18]. The syndrome could be partly due to brain damage caused by repeated acute hypoglycemia or chronic hyperammonemia. However, focal or multifocal lesions of hypoglycemic brain injury are not typical in patients with this syndrome. Possible mechanisms could be as follows: 1) Some researchers believe that increased GDH activity in the brain is the main cause of neurological impairment. GDH hyperactivity leads to increased consumption of glutamate, resulting in reduced synthesis of GABA and disrupting the balance in neurotransmission [19]; 2) There are also studies showing that GDH may interfere with the synthesis of amino acid-related neurotransmitters such as glutamate and GABA [19, 20]; 3) Another mechanism proposed by Komlos et al. is that GDH overactivity in the brain promotes the formation of astrocytes, resulting in an imbalance between glial and neuronal cells that may lead to seizures and severe developmental defects [5].

In summary, development defects in the CNS in GDH-HI patients could be caused by multiple factors, including chronic hyperammonemia, and hyperinsulinemia, the more detailed mechanisms need to be further studied.

### Relationship between GLUD1 genotypes and neurological clinical phenotypes

Neurological symptoms in children with GDH-HI mainly manifest as epilepsy. In reports published by Kapoor [21], Bahi-Buisson [18], Raizen [19], and Diao [22], all children with the p.S445L mutations (6 cases in total) developed generalized tonic-clonic seizures between the first week and fifth month after birth [23].

A study in 2018 by Bahi-Buisson et al. from France proved [18] that mutations in exons 6 and 7 of *GLUD1* (81%), which encode the GTP binding sites, were more likely to cause epilepsy than mutations in exons 11 and 12 (25%), which encode the hinge and antenna-like domains. Among patients with mutations in exons 6 and 7, 41% and 56% of them displayed epilepsy, respectively. However, the position of mutation sites does not affect the occurrence probability of other neurological disorders, especially learning disorders. More than 77% of children with GDH-HI have a mild to moderate learning disability, which is often associated with seizures. So far, there is no evidence showing a correlation between blood ammonia levels and epilepsy or mental retardation.

In 2016, Reizen et al. found that children with GDH-HI carrying the p.R221C mutations were prone to mild cognitive impairment, ADHD, and refractory epilepsy [23]. Patients with the p.R221C mutations usually have a late onset (7–23 months). Some are even asymptomatic until adulthood. However, some reports showed that p.R221C mutations could lead to early onset at 10 weeks and 3 months after birth. Patients with first symptoms of hypoglycemia were at higher risk of epilepsy and cognitive impairment. The study also found that children carrying the p.R221C mutations had a higher chance to develop absence seizures and eyelid myoclonic seizures at the age of 2 to 6 years old. Additionally, 40% of these cases were not responsive to anti-epileptic treatment [23].

## Clinical presentation of GDH-HI

Children with GDH-HI typically have a normal birth weight and rarely demonstrate evidence of hypoglycemia in the neonatal period. The average age of onset is 4 months and the median age of onset is 11 months. However, onset can range as early as the neonatal period up to 24 months of life [15]. In rare cases, individuals are not diagnosed until adulthood.

Clinical features of children with GDH-HI include persistent mild hyperammonemia, hypoglycemia after fasting or high protein (especially leucine) diets, and neuroglycopenic symptoms including seizures. Symptoms tend to be milder than in other types of CHI, manifesting as unprovoked convulsions, low muscle tone, a decline of response, drowsiness, etc.

Most children with GDH-HI have hyperammonemia, with blood ammonia levels are 2–5 times above the upper limit of normal. Very few patients might have normal blood ammonia levels (only 2 cases were reported so far) [21, 24]. The blood ammonia levels in children with GDH-HI are not as high as Urea cycle disorder (UCD) patients, and the blood ammonia level is stable. The blood ammonia levels in children with GDH-HI will not increase significantly due to protein intake and are not related to blood glucose levels as well [12].

Reizen et al. [23] reported that GDH-HI patients have a higher rate of epilepsy. In some case reports, ~ 43%, 46%, and 64% of children with GDH-HI were associated with epilepsy, respectively. Epilepsy usually occurs months or years after the onset of HI. Based on different studies, the median age of seizures is between 4 and 11 months. Seizures in these patients are mostly atypical absence seizures and eyelid myoclonic seizures. Very few patients show generalized tonic-clonic seizures and localized motor seizures. The occurrence of these seizures can be spontaneous or induced by light stimulation. EEG patterns of these patients were similar to those with regular epilepsy. During a seizure, the EEG may show irregular ratchet discharge with high amplitude. Epilepsy in GDH-HI patients is typically insensitive to anti-epileptic drugs.

Patients with GDH-HI may also have other types of neurological abnormalities. Studies showed that ~ 50–80% of patients display mental retardation and learning disabilities, and these risks may be higher in GDH-HI patients with epilepsy. Some children with both GDH-HI and epilepsy may also have behavioral problems, such as social disorders, attention deficit hyperactivity disorder (ADHD). In rare cases, these patients might also have ataxia and dystonia.

According to previous reports, most GDH-HI patients did not display abnormalities in neurological imaging. However, a small number of patients may display abnormalities durin1g brain magnetic resonance imaging (MRI), such as left frontal encephalomalacia, cerebral atrophy, porencephalic cysts, and left frontal brain cysts [18]. Studies demonstrated that these patients show a higher incidence and earlier onset of neurological syndromes compared to patients with other types of CHI. Therefore, early diagnosis and treatment are particularly important to prevent long-term neurological complications.

## Diagnosis of GDH-HI

Expeditious diagnosis of CHI will allow prompt and appropriate treatment and reduce the risk of permanent brain damage from persistent and recurrent severe hypoglycemia. Diagnosis of CHI is based on the clinical features of children and their biochemical markers during hypoglycemic onset. Specimens should be obtained at the time of spontaneous presentation and before treatment whenever possible. These biochemical markers mainly include 3 types: non-ketogenic hypoglycemia; excessive secretion of insulin when blood glucose levels are low is required to increase the amount of glucose to avoid the occurrence of hypoglycemia. The commonly used diagnostic criteria are the criteria recommended by Ferrara et al. [25] in 2016. These criteria are as follows: when intravenous plasma blood glucose level is lower than 2.8 mmol/L, there is still insulin secretion (serum insulin can still be detected with a level higher than 1–2 μU/mL, or plasma C-peptide can be detected with a level higher than 0.2 mmol/L), low plasma free fatty acids (< 1.7 mmol/L), low levels of β-hydroxybutyric acid (< 1.8 mmol/L), positive for glucagon stimulation test (intramuscular or intravenous injection of glucagon at a dose of 0.5 mg for newborns and a dose of 1 mg for older children, following with a plasma glucose increase ≥ 1.7 mmol/L within 40 min is regarded as positive).Those with mild symptoms of hypoglycemia may take the diagnostic fasting test for diagnosis.

Genetic mutation testing is now well established as the standard of care to define the best approaches to the treatment of CHI. Molecular diagnosis helps to identify the causative genes. This helps with management and understanding the long-term prognosis. In some cases, next-generation sequencing is required to excluded intronic mutations. Parental origin testing should be included when patient testing [26]. Those with hyperammonemia and prone to postprandial hypoglycemia are likely to be diagnosed with GDH-HI [27]. If GLUD1 mutations were identified by genetic examination, the children could be diagnosed as GDH-HI.

## Histological types of GDH-HI

Depending on the histological features of the pancreas in children, CHI can be classified into three types: focal, diffuse, and atypical. Current studies show that the pancreatic histological type of GDH-HI is diffuse, where the pancreatic β-cells show increased nuclear size throughout the pancreas [28].

## Treatment approaches

The main treatment methods for GDH-HI include diazoxide treatment and protein-restricted diets. As a potassium channel activator, diazoxide binds to the sulfonylurea receptor 1(SUR1) subunit and inhibits closure of the K_ATP_ channel with subsequently diminished insulin secretion by pancreatic β cells. Thus, diazoxide is used as the main and preferred drug for CHI treatment.

The recommended dose range for diazoxide is 5 to 15 mg/kg/day in 2 or 3 divided doses. The starting dose should be selected according to the risk profile of the neonate (lower starting dose for children with cardiac disease). The dose is adjusted to the plasma glucose level, maximum dose not exceeding 15 mg/kg/day [29]. A dose reduction is required if hyperglycemia occurs. The potential risk of hyperglycemia increases at the higher dose range of 15 mg/kg/day when the serum concentration of diazoxide exceeds 100 μg/mL [30].

The most common side effect of diazoxide is hypertrichosis, and the most serious but rare side effect is pulmonary hypertension. Other adverse effects included fluid retention, pulmonary edema, neutropenia, thrombocytopenia, hyperuricemia, and hyperglycemia [29]. Before diazoxide treatment, the cardiopulmonary health of children should be carefully evaluated. Thiazide diuretics should be begun as soon as diazoxide is started to prevent the side effects of fluid retention, with hydrochlorothiazide at 1 to 2 mg/kg/day in two divided doses. Complete blood counts (CBC) and uric acid levels should be measured at baseline and 5 to 7 days after starting diazoxide therapy, and thereafter every 3–6 months for CBC and every 6 months for uric acid levels [29].

The efficacy of diazoxide should be evaluated 5 days after treatment on maximum dose by a fasting challenge [31]. Children are generally defined as being responsive to diazoxide if plasma glucose concentrations maintain > 70 mg/dL (3.9 mmol/L) during a prolonged fast appropriate for age or hyperketonemia (plasma β-hydroxybutyric acid > 2.0 mmol/L) is observed before the plasma glucose concentration is ≤ 50 mg/dL (2.8 mmol/L) on diazoxide [32]. It indicates unresponsiveness to diazoxide if plasma glucose concentrations is not maintained above 70 mg/dL (3.9 mmol/L) during the fasting challenge [33].

For children with GDH-HI, a protein-restricted diet is recommended as an adjunct therapy, i.e., restrict leucine to less than 200 mg per meal. Restraining protein levels can help prevent postprandial hypoglycemia in children with GDH-HI and effectively reduce diazoxide dosage for the primary treatment [34].

As the KATP channel of islet β cells is normal in children with GDH-HI, diazoxide can effectively control the hypoglycemia of GDH-HI children, but is ineffective for hyperammonemia. Benzoate or N-carbamoyl glutamate also fails to reduce blood ammonia levels in patients with GDH-HI [12]. Currently, there are no effective intervention therapies for hyperammonemia in GDH-HI patients. Finding effective GDH inhibitors may be a more ideal option. Currently, few GDH inhibitors have been reported. The most commonly reported inhibitor of GDH is EGCG. EGCG is effective in inhibiting GDH, but has not been used clinically due to poor intestinal absorption, instability in solution and low bioavailability [4].

Ebselen is an anti-inflammatory compound in Phase III clinical trials and has been reported for many therapeutic applications, such as intestinal anti-inflammatory effects, neuroprotective effects and anti-tumor activities [35]. Recent studies have identified Ebselen as a reversible active site inhibitor of GDH and a potentially valuable drug for GDH-HI patients. This study is still in the early stage, and there is a lack of safety and toxicology studies on the drug for GDH-HI patients [36].

### Prognosis of GDH-HI

Hypoglycemic episodes in most children with GDH-HI can be controlled through diazoxide treatment and protein-restricted diets. These children typically do not require surgery and display a good prognosis.

As the disease progresses, a small number of children may show spontaneous remission. The criteria for spontaneous remission of GDH-HI are as follows: for those who take diazoxide orally, after discontinuation of diazoxide for at least 5 days and fasting for 18–48 h (depending on age), the blood glucose levels maintained at 70 mg/dL (3.88 mmol/L) or above, or hyperketonemia (plasma β-hydroxybutyric acid > 2.0 mmol/L) is observed [37].

Due to untimely diagnosis, a significant number of children with GDH-HI displayed neurological sequelae, including mental retardation, cognitive impairment, learning disability, and social disorder [38]. The prognosis of these neurological syndromes was not related to blood ammonia levels. One of the largest cohort studies of GDH-HI patients monitoring neurological prognosis [23] showed that children who displayed a symptom within the first 3 months after birth had light to mild developmental retardation and had a seizure occurrence rate higher than 70%. Poor compliance to diazoxide treatment can lead to exacerbation of epilepsy. Whether the frequency and severity of episodes of hypoglycemia affect neurological prognosis remains to be studied further. In summary, the neurological prognosis of GDH-HI patients depends on multiple factors, including the age of onset, diet control, and compliance to medication. Early diagnosis and treatment are extremely important to avoid long-term neurological complications. Children with GDH-HI should regularly test blood glucose, take measures to control low blood glucose levels as early as possible, and visit the doctor regularly to ensure proper drug safety and dose.

## Conclusions

GDH-HI is the second most common form of CHI, characterized by fasting hypoglycemia and hypoglycemia induced by high-protein diets. The patients usually develop hyperammonemia as well. Patients are responsive to diazoxide treatment. Some patients may have neurological sequelae. The occurrence rate of these neurological syndromes is higher in GDH-HI patients compared with patients bearing other types of CHI. With new research ongoing, effective GDH inhibitors will be developed which hopefully will provide better therapeutic effects for children with GDH-HI.

## Data Availability

Orhpant journal of rare disease remains neutral with regard to jurisdictional claims in published maps and institution affiliations.

## Abbreviations

CHI: Congenital hyperinsulinism
GDH-HI: Glutamate dehydrogenase hyperinsulinism
GDH: Glutamate dehydrogenase
GLUD1: Glutamate dehydrogenase 1
GLUT2: Glucose transporter 2
GCK: Glucokinase
G6P: Glucose-6-phosphate
TCA: Tricarboxylic acid
KATP: ATP-sensitive potassium
GTP: Guanosine triphosphate
NADH: Nicotinamide adenine diphosphate hydride
ADP: Adenosine diphosphate
ATP: Adenosine triphosphate
HI/HA: Hyperinsulinism/hyperammonemia
HHF6: Familial hyperinsulinemic hypoglycemia-6
α-KG: Alpha-ketoglutaric acid
DES: Diethylstilbestrol
EGCG: Epigallocatechin gallate
SIRT4: Silent information regulator 4, Sirtuin 4
MCD: Malonyl coenzyme A decarboxylase
NAG: N-acetylglutamate
CPS: Carbamoyl phosphate synthetase
BCH: 2-Aminobicyclo [2, 2, 1] heptane-2-carboxylic acid
GABA: γ-Aminobutyric acid
